# Whole genome sequence analysis of *Neisseria meningitidis* strains circulating in Kazakhstan, 2017–2018

**DOI:** 10.1371/journal.pone.0279536

**Published:** 2022-12-28

**Authors:** Alexandr Shevtsov, Zabida Aushakhmetova, Asylulan Amirgazin, Olga Khegay, Dinara Kamalova, Bibiaisha Sanakulova, Askar Abdaliyev, Dinagul Bayesheva, Aliya Seidullayeva, Yerlan Ramankulov, Alexandr Shustov, Gilles Vergnaud

**Affiliations:** 1 National Center for Biotechnology, Astana, Kazakhstan; 2 National Centre of expertise CSEC MN RK, Astana, Kazakhstan; 3 Medical University Astana, Astana, Kazakhstan; 4 Multidisciplinary City Children’s Hospital №3, Astana, Kazakhstan; 5 School of Science and Humanities Nazarbayev University, Astana, Kazakhstan; 6 CEA, CNRS, Institute for Integrative Biology of the Cell (I2BC), Université Paris-Saclay, Gif-sur-Yvette, France; Massey University, NEW ZEALAND

## Abstract

*Neisseria meningitidis* (meningococcus) is a cosmopolitan bacterium that is often found in the upper respiratory tract of asymptomatic humans. However, *N*. *meningitidis* also causes meningeal inflammation and/or sepsis in humans with a periodic resurgence in incidence and high mortality rates. The pathogen is highly diverse genetically and antigenically, so that genotyping is considered important for vaccine matching to circulating strains. Annual incidence of meningococcal disease in Kazakhstan ranges between 0.2 and 2.5 cases per 100 thousand population. In total, 78 strains of *N*. *meningitidis* were isolated from clinical patients and contact persons during the years 2017–2018 in Kazakhstan. Of these, 41 strains including four from the patients and 37 from contacts, were sequenced using Illumina MiSeq. *In silico* typing was completed using the Neisseria pipeline 1.2 on the Galaxy Workflow Management System and PubMLST. Whole genome SNP (single nucleotide polymorphisms) trees were built using BioNumerics 8. Seven-gene multilocus sequence typing (MLST) identified ten sequence types (ST), two of which have not been previously described (ST-16025; ST-16027). ST-16025 was detected in two patients with invasive meningococcal disease in 2017 and 2018 in Akmola region and 16 contacts in 2017 in Turkistan region. This prevalent type ST-16025 demonstrates considerable intertypic diversity as it consists of three subcomplexes with a distance of more than 2000 SNPs. Invasive and carrier strains belong to different serogroups (MenB and MenC), PorA and FetA_VR. Two invasive strains were MenB, one MenC and one MenW (Hajj lineage). The strains from the contact persons were: MenC (n = 18), cnl (n = 9), MenY (n = 7), MenW (n = 1), MenB (n = 1) and one unidentifiable. Different numbers of alleles were present: 12, 11, 7, and 7 alleles for PorA, FetA, fHbp, and NHBA, respectively. This study is the first report of the genetic diversity of *N*. *meningitidis* strains in Kazakhstan. Despite limitations with the studied sample size, important conclusions can be drawn based on data produced. This study provides evidence for regulatory authorities with regard to changing routine diagnostic protocols to increase the collecting of samples for WGS.

## Introduction

*Neisseria meningitidis* (meningococcus) is a Gram-negative bacterium with a spherical cell appearance (cocci). During non-epidemic time, about 10% of the population carry *N*. *meningitidis* in the upper airways asymptomatically [1, 2]. The proportion of carriers tends to be larger in population groups with close communal contacts such as military personnel, students, as well as in those having occupational or domestic contacts with patients with meningococcal infection [3–5]. Meningococcal carriage is an immunizing process, resulting in a systemic protective antibody response against capsular antigens [6, 7].

In rare cases, meningococci penetrate the mucosal epithelium and enter the bloodstream, leading to sepsis. Meningococci can then cross the blood-brain barrier and spread into the central nervous system (CNS) causing meningitis. Invasive meningococcal disease (IMD) typically involves meningococci from six out of the 12 currently defined serogroups: A, B, C, W, X, and Y [8]. IMD is characterized by a rapid onset and poor outcome, with a mortality rate of ~80% if untreated and 4–20.0% if treated with appropriate antibiotics [9]. Incidence ranges from a few sporadic cases in developed countries (less than 0.1 per 100 thousand), to about 1/1000 in the so-called meningitis belt in North Africa [2, 10]. The world-wide prevalence of IMD is difficult to estimate because of differences in diagnostic approaches and surveillance [11]. The gold standard is isolation of pure bacterial cultures followed by serotyping of the isolated strain [11]. Progress in typing of strains has greatly evolved upon introduction of genetic methods. Having started with MLST [12, 13] and currently widely utilizing whole-genome sequencing (WGS), the genetic methods provide a wealth of information not only for epidemiological surveillance but also to study the pathogen’s biology and population structure. WGS has the highest discriminatory ability and provides invaluable information on genetic relationship and antigenic variability. WGS can also assist vaccines selection and help evaluate the effectiveness of vaccination [2, 14].

Analysis of cases of meningococcal infection in Kazakhstan over a twenty-year period from 2001 to 2020 shows a decrease in the incidence. The average incidence from 2001 to 2010 was 1.87 cases per 100 thousand population, whereas from 2011 to 2020 the average incidence was 0.72 cases per 100 thousand population. Increases in the incidence rate are cyclical and observed every 3–6 years. In the 2001–2020 timeframe, such increases were recorded in 2002 (2.5 cases per 100 thousand), 2007 (2.2 cases per 100 thousand) and 2015 (2.45 cases per 100 thousand). The incidence over the past 6 years was, in 2016: 0.68/100,000 (number of diagnosed cases, n = 120), in 2017: 0.35/100,000 (n = 62), in 2018: 0.53/100,000 (n = 91), in 2019: 0.34/100,000 (n = 63), in 2020: 0.19/100,000 (n = 36) and in 2021: 0.11/100,000 (n = 22). Numbers of deaths: 2015–21 cases, 2016–7 cases, 2017–11 cases, 2018–15 cases, 2019–6 cases, 2020–7 cases and in 2021–5 cases. In 2017, the majority of cases were registered in Akmola and Turkistan regions (16 and 12 cases, respectively); in 2018, a sharp rise in the incidence was observed in the Almaty region (56 cases). The lowest incidence rate was observed in years 2020–2021 and might be an outcome of restrictive measures imposed by the COVID19 pandemic. The epidemiologic surveillance system collects data on IMD cases and contacts, but to the best of our knowledge no studies have been conducted in Kazakhstan to estimate the carrier state rates in the general population. No data on genotypes of *N*. *meningitidis* circulating in Kazakhstan and sequence diversity of vaccine-related genes have been available before this study. This data deficiency makes impossible vaccine matching. This study presents preliminary albeit novel data on the genetic diversity of meningococcal strains isolated in Kazakhstan. All reported genotypes were inferred from WGS data.

## Materials & methods

*N*. *meningitidis* strains were collected using approved protocols and rules for the diagnosis and control of meningococcal infections in Kazakhstan. Human samples (cerebro-spinal fluid CSF and oropharyngeal swabs) and associated strains were anonymized. This retrospective study was approved by the local ethics committee of the National Center for Biotechnology (Protocol No. 5 dated 16.10.2020). The IRB has waived the obtaining of informed consent, based on the grounds that: 1. the studied strains are archived, and had been deposited in the museum without any link to the source person. 2. the authors of this work were not involved in the collection of the strains. 3. the strains described in the manuscript are anonymous with no identifiers of patients. 4. all subjects from which the samples were collected had given written informed consent, in the medical organization providing the treatment or epidemiological investigation. 5. this study will not adversely affect the rights and welfare of human subjects.

### Isolation, identification and selection of *N*. *meningitidis* for WGS

During 2017–2018, the State Epidemiological Service investigated CSF samples from patients with meningococcal infections and oropharyngeal swabs from persons having had a close contact with patients in five regions of Kazakhstan. In total, 74 cultures were produced from oropharyngeal swabs collected from contact persons, and four additional cultures were produced from patients.

Isolation of meningococcus was carried out by inoculation in a nutrient broth for cultivation and isolation of meningococci (FBIS SRCAMB, Obolensk, Russia) with 20% bovine serum and Columbia sheep blood agar (Srichem). Bacterial cultures were incubated for 24 hours at 37°C in an atmosphere of 5% CO2. Low isolation rates were observed in patients with a history of antibiotic therapy prior to sampling and diagnostic protocols. For each successful culture, four colonies were subcultured using the same conditions. The presence of meningococci was confirmed by microscopy, latex beads agglutination (Pastorex Meningitis, BioRad), testing for oxidative and catalase activity, and sequencing of the *16S rRNA* fragment. Genogrouping typing was performed by multiplex polymerase chain reaction (PCR) [9].

In Kazakhstan, a diagnosis of IMD requires a confirmation by a positive PCR from CSF/blood. Alternatively, isolation of a pure culture of *N*. *meningitidis* may be used for the confirmation. Due to the simplicity and short time of the PCR diagnostics, it is predominantly used, whereas the culture isolation is performed in 1–10% of cases. On the contrary, an epidemiological investigation of contact persons requires the isolation of pure cultures from the pharynx with subsequent typing. Because of the infrequent use of the culture isolation, during 2017 and 2018, only four strains had been isolated from patients with IMD. At the same time, 74 strains were collected from contact persons. All strains from patients with IMD were taken for WGS, and 37 strains from contact persons were used for WGS. When selecting strains from contacts, the data of association with IMD patients were taken into account, at least one sample from contact with the patient was included in the WGS (S1 Table).

### Whole genome sequencing, in silico typing and phylogenetic analysis

Isolation of genomic DNA from bacteria was performed using a QIAamp DNA Mini Kit (Qiagen, USA) according to the manufacturer’s instructions. DNA libraries for sequencing were prepared using the Nextera XT DNA Library Preparation Kit (FC-131-1024, Illumina, San Diego, CA, USA) according to the manufacturer’s instructions. Sequencing was performed on Illumina MiSeq sequencer using the MiSeq Reagent Kit v3 600 Cycles (MS-102-3003, Illumina, San Diego, CA, USA). Data pre-processing and quality control was performed as previously described [11]. In silico typing was completed using the Neisseria pipeline 1.2 on the Galaxy Workflow Management System [11]. MLST was based on the MLST *Neisseria spp*. scheme with seven loci hosted by PubMLST [13]. Whole genome SNP (wgSNP) analysis was performed using BioNumerics by mapping reads on reference genome assembly accession GCF_000009105 (strain Z2491) or M07149 assembly accession GCF_001697425 as stated. wgSNP trees involving strains from diverse WGS lineages [15] were drawn using the Bio-Neighbor Joining (BIONJ) algorithm [16]. Trees involving a single ST were drawn using Maximum Parsimony. Clustered SNPs indicative of recombination events were detected using Gubbins version 2.4.1 [17]. Genome assemblies were deposited in the BIGSdb hosted at https://pubmlst.org/ [13]. Genomes in BIGSdb with similar core genome MLST were identified using the Genome comparator tool.

## Results

### Strain clustering based on WGS data analysis

**Fig 1:**
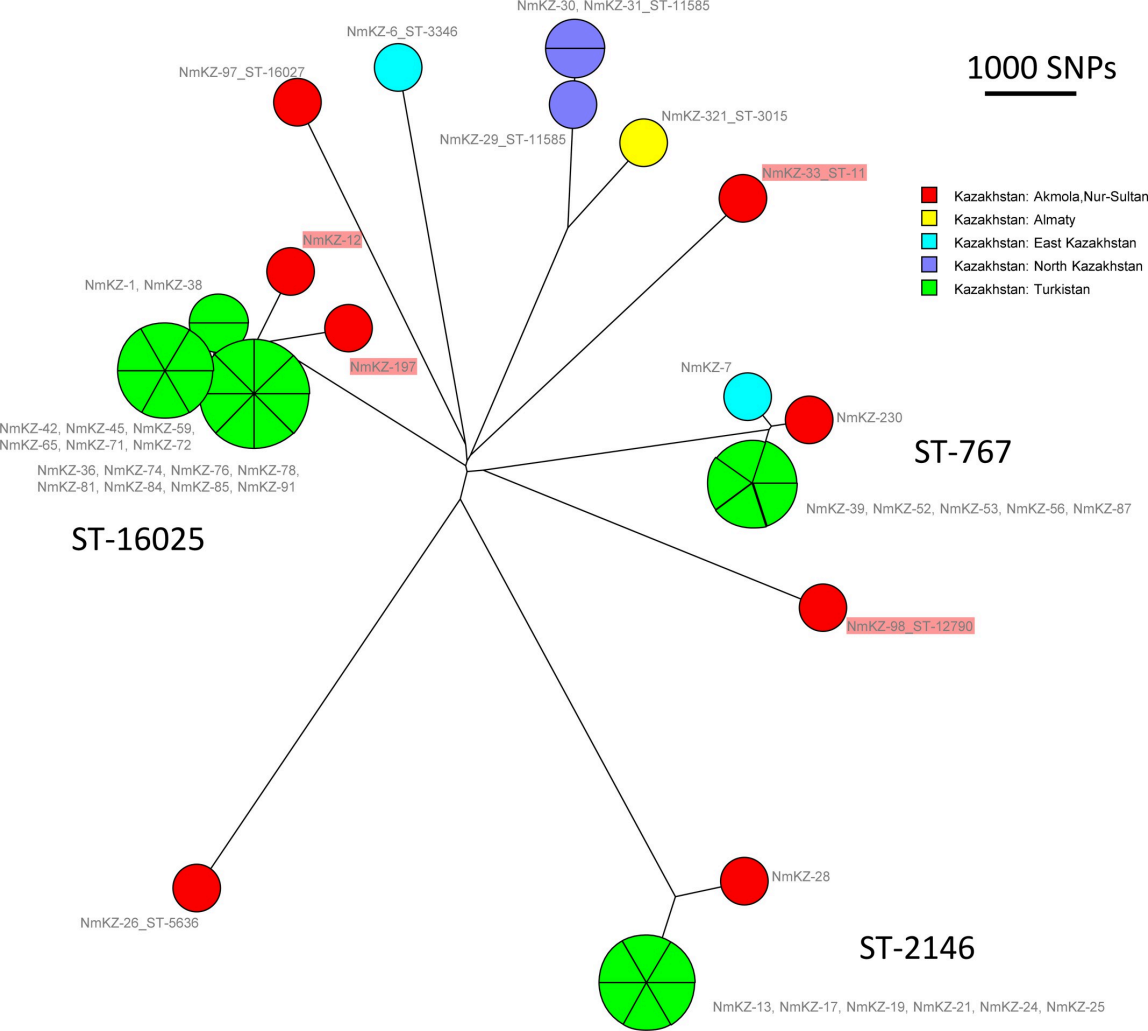
Bio-Neighbor Joining tree of the 41 Kazakhstan strains collected in 2017–2018. SNPs (32318) were called by mapping on reference genome Z2491 assembly accession GCF_000009105. Coloring reflects region of origin. Strains are labeled with strain Id and MLST7 ST. Four strains recovered from CSF are boxed in light red.

Ten sequence types (ST) were identified among the 41 strains (Tables 1 and S1). Fig 1 shows the result of a clustering analysis of the 41 sequenced strains based on wgSNP. The clustering is congruent with MLST7 assignment. Four STs are represented by varying numbers of strains (3–18) and in total comprise 35 strains. The remaining six STs are represented by single strains. Two among the ten STs are new profiles combining known alleles. Eighteen strains with new MLST ST-16025 were identified in Turkistan and Akmola regions during 2017–2018. Four MLST7 STs in the PubMLST database show a one-locus difference with this new profile. Two of these are represented by strains from Russia. New profile ST-16027 was observed in one strain. The closest MLST7 profile ST-2957 represented by a single strain in PubMLST differs at two loci.

**Table 1:** Genetic and antigenic characteristics of isolated strains *N*. *meningitidis*.

Sequence type	Date collection (mm.year)	Region	Number of strains	Serogroup	Clonal complex	PorA	FetA_VR	NHBA peptide	fHbp peptide (Novartis variant/Pfizer subfamily)
invasive									
ST-11	11.2017	Akmola	1	W	cc11	P1.5,2–2	F1-1	96	613 (1/B)
ST-12790	06.2018	Akmola	1	C	cc4821	P1.22,2–14	F1-3	9	1166 (2/A)
ST-16025	06.2017	Akmola	1	B		P1.0,25–16	F3-9	20	706 (1/B)
ST-16025	06.2018	Akmola	1	B		P1.5–1,2–2	F1-90	20	706 (1/B)
carrier									
ST-11585	08.2017	North Kazakhstan	1	unidentifiable		P1.5–3,10–4	F3-72	20	1166 (2/A)
ST-11585	08.2017	North Kazakhstan	2	cnl		P1.5–3,10–4	F3-72	20	1166 (2/A)
ST-16025	12.2017	Turkistan	1	C		P1.22,13–2	F3-10	20	706 (1/B)
ST-16025	01.2017	Turkistan	15	C		P1.22,13–2	F3-9	20	706 (1/B)
ST-16027	04.2018	Akmola	1	C	cc364	P1.22,2–26	F3-6	6	43 (2/A)
ST-2146	06.2017	Turkistan	6	cnl	cc198	P1.18,2–25	F5-5	10	614 (3/A)
ST-2146	08.2017	Akmola	1	cnl	cc198	P1.18,2–25	F5-70	10	614 (3/A)
ST-3015	06.2018	Almaty	1	W		P1.18–1,2–3	F3-4	20	1166 (2/A)
ST-3346	03.2017	East Kazakhstan	1	B	cc41/44	P1.21,2–4	F1-66	188	849 (2/A)
ST-5636	07.2017	Akmola	1	C		P1.22,23–7	F3-9	688	849 (2/A)
ST-767	06.2018	Akmola	1	Y	cc167	P1.5–1,10–8	F1-3	9	1084 (2/A)
ST-767	12.2017	Turkistan	5	Y	cc167	P1.5–1,10–8	F1-3	9	1084 (2/A)
ST-767	03.2017	East Kazakhstan	1	Y	cc167	P1.5–1,10–8	F1-3	9	1084 (2/A)

**Fig 2:**
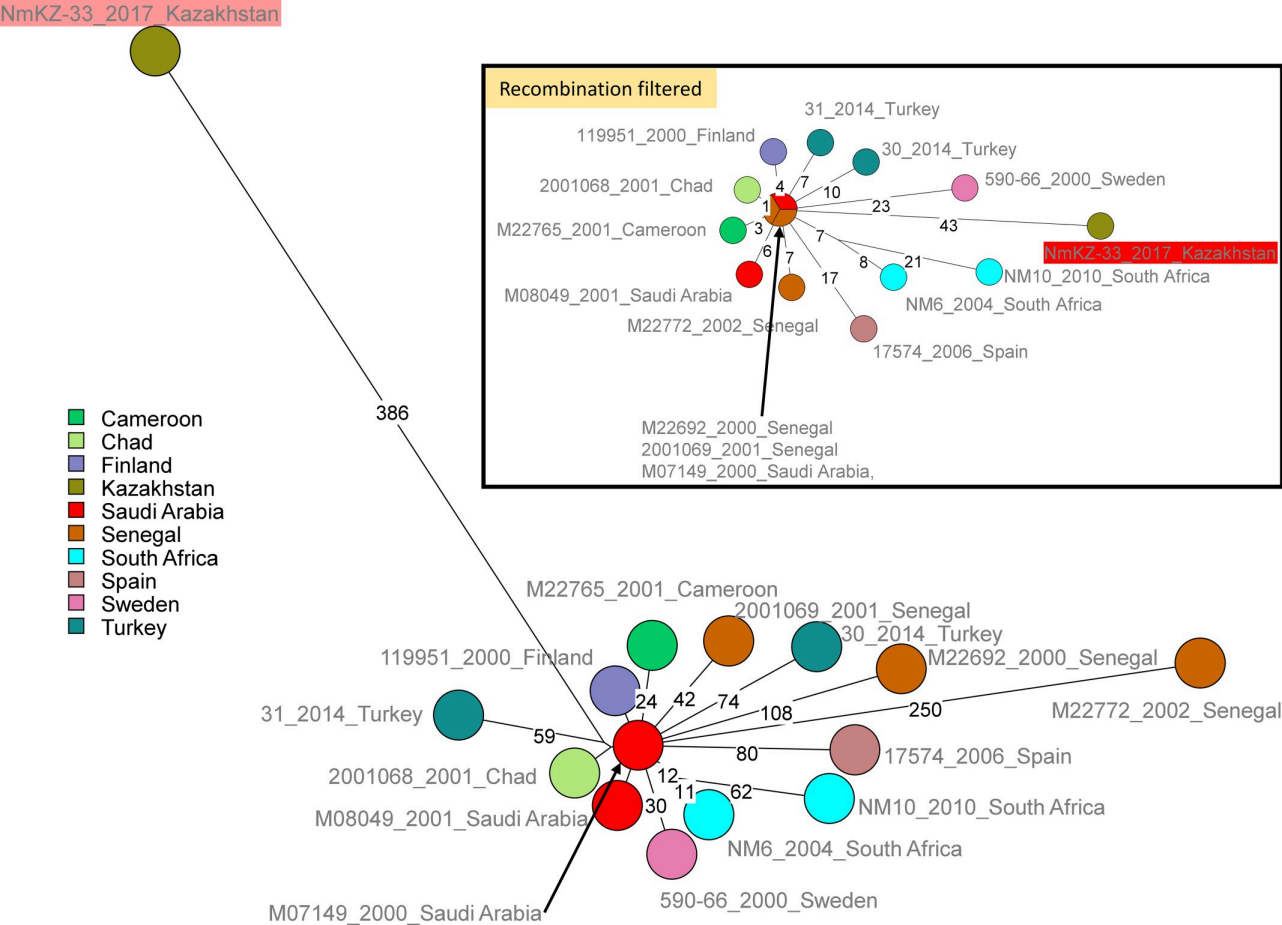
Comparison of the ST11 invasive strain NmKZ-33 from Kazakhstan with 14 PubMLST nearest neighbors. A representative subset among strains with a cgMLST distance lower than 150 to Kazakhstan strain NmKZ-33 is shown. 1153 SNPs were called by mapping on genome M07149 assembly accession GCF_001697425. The size of the resulting maximum parsimony tree is 1162. Branch lengths above 10 SNPs are indicated. Strains are labeled with strain Id, year of isolation and country, and colored according to country of origin. Recombination-filtered inset: 157 SNPs are retained after filtration with Gubbins [17]. The inset shows the associated Maximum Parsimony Tree (tree size 157).

The two IMD strains have been assigned to new MLST type ST-16025, one strain to ST-12790 and one strain to ST-11. The pubMLST nearest neighbors of the invasive ST-11 strain were searched among the 3789 pubMLST strains with cgMLST data belonging to cc11 capsule group W (last accessed 26/10/2021). Invasive ST-11 strain NmKZ-33 isolated in 2017 is closest to strains isolated in year 2000 in Saudi Arabia, known as the Hajj lineage. These closest neighbors differ at 105–110 loci in terms of cgMLST. Fig 2 illustrates the output of a wgSNP clustering of representative close neighbors, including strains differing at up to 150 cgMLST loci. NmKZ-33 displays the longest branch. Almost 90% of the corresponding SNPs are recognized as clustered by Gubbins analysis. Seventeen potential recombined segments covering a total of 67 kb are identified. The inset in Fig 2 shows the clustering obtained after the masking of blocks of SNPs.

### Horizontal genetic transfer events within the main clusters

**Fig 3:**
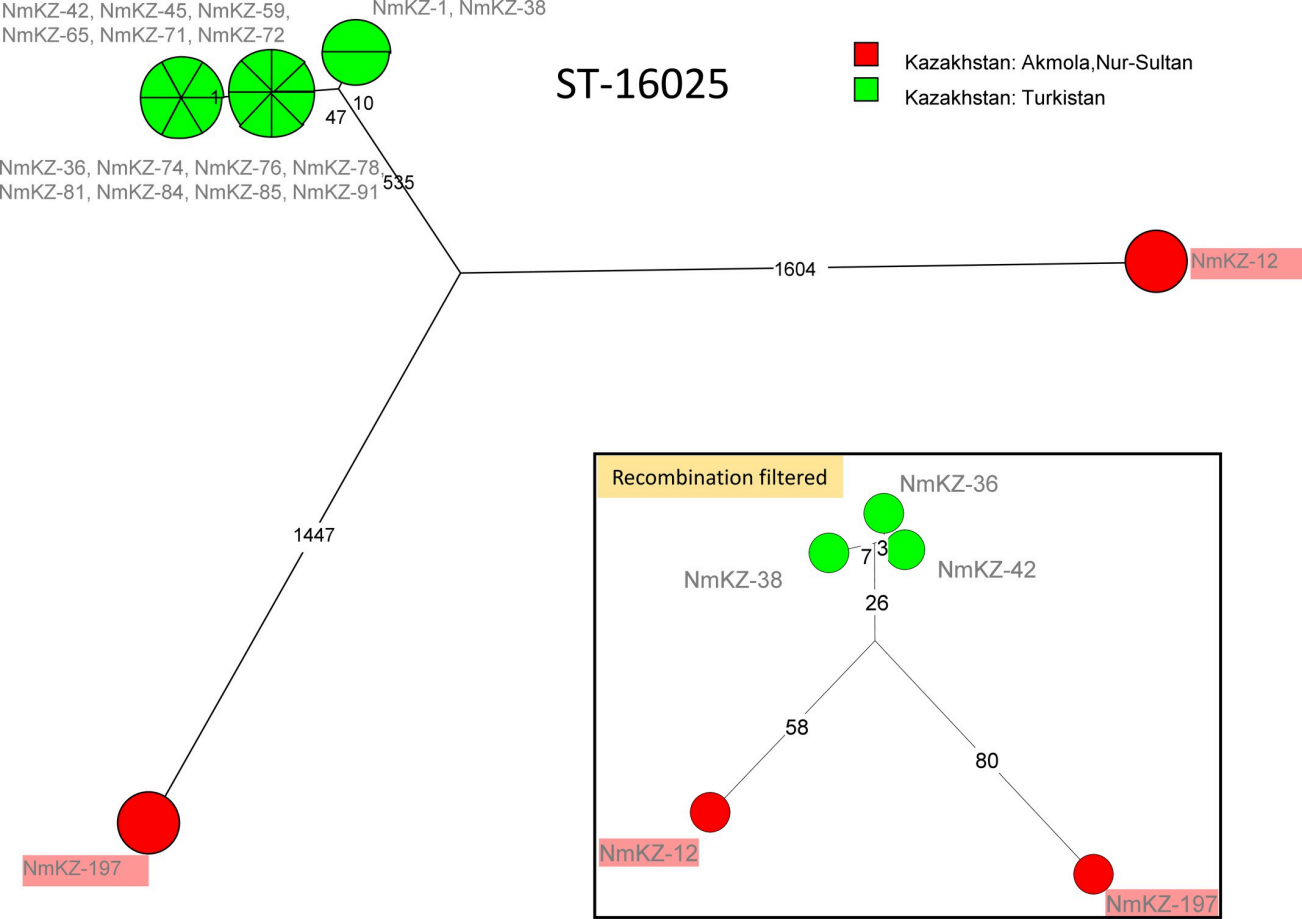
Focus on ST-16025. Eighteen strains were assigned to the newly described ST-16025. Strains are colored according to region of origin. Two invasive strains are boxed in light red. SNPs (3635) were called by mapping on reference genome Z2491 assembly accession GCF_000009105. The size of the resulting Maximum Parsimony Tree is 3644. Branch lengths are indicated. “Recombination filtered” inset: 176 SNPs are kept after filtering of clustered SNPs by Gubbins. No homoplasia in the Maximum Parsimony tree.

**Fig 4:**
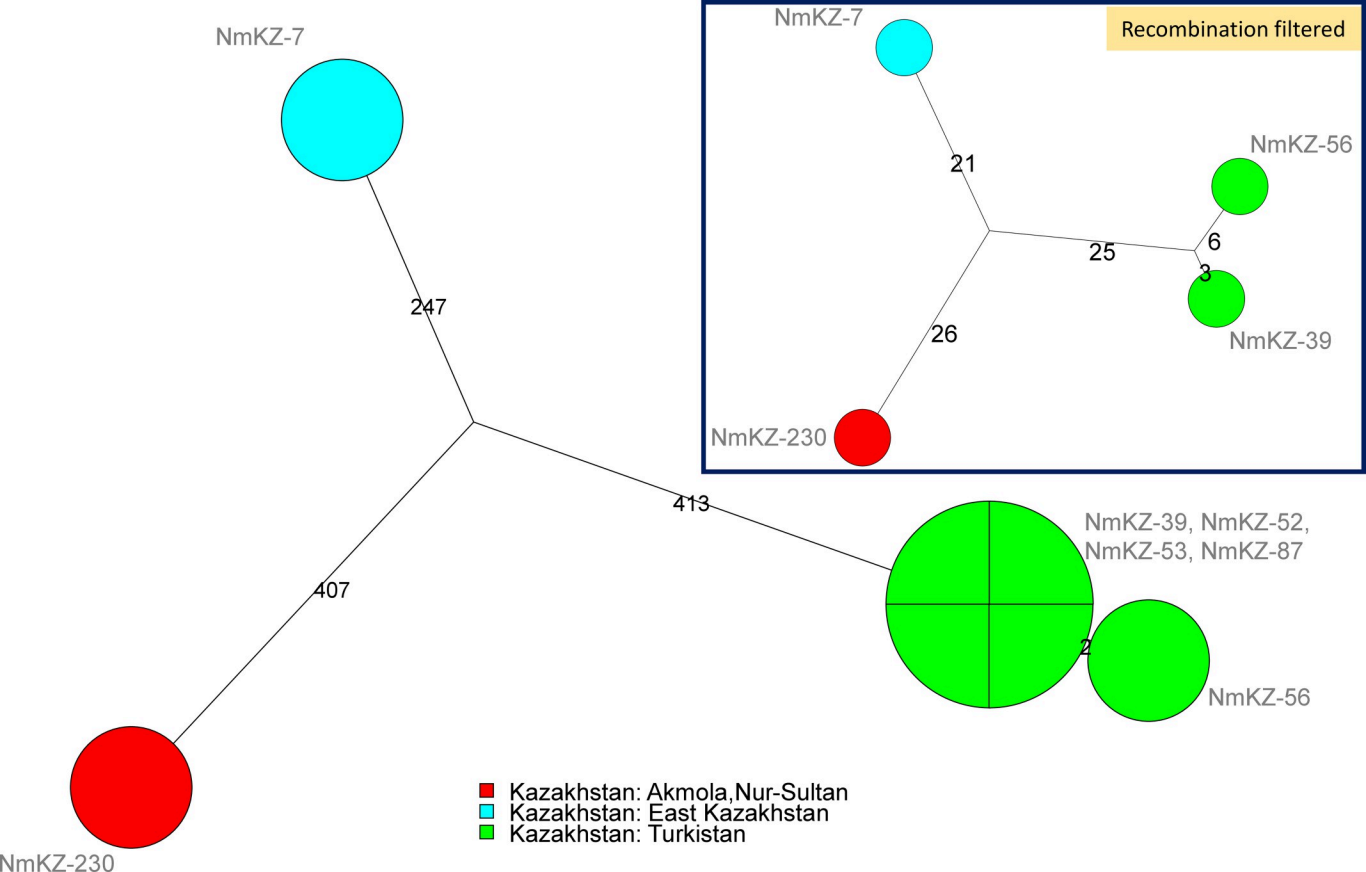
Focus on ST-767. Seven strains were assigned to ST-767 (cc167). Strains are colored according to region of origin. SNPs (1069) were called by mapping on reference genome Z2491 assembly accession GCF_000009105. The size of the resulting Maximum Parsimony Tree is 1069 (no homoplasia). Branch lengths are indicated. “Recombination filtered” inset: 80 SNPs are kept after filtering of clustered SNPs by Gubbins, Maximum Parsimony tree size, 81.

wgSNP analysis of the 18 strains with new MLST profile ST-16025 separates the cluster into three branches (Fig 3). The main one corresponds to 16 carrier strains collected in 2017 in Turkistan region. The two other branches are constituted by the two invasive strains, NmKZ-12 recovered in June 2017 and NmKZ-197 obtained in June 2018 in Akmola region. Subcomplexes differ from each other by more than 2000 SNPs. Fifty regions of high SNP density span 450 kb of the genome. The inset in Fig 3 shows the result of clustering analysis obtained after masking these regions. Despite their identity in MLST7, the invasive strains show different phylogenetic clustering between each other and from the non-invasive strains. Considerable differences in capsular antigen have been predicted from the gene sequences. Fig 4 illustrates this for ST-16025.

### In silico prediction of serogroup, PorA and FetA subtypes

One strain with ST-11 (clonal complex cc11, serogroup W) and ST-12790 (cc4821, serogroup C) were isolated from CSF samples (S1 Table). ST-767 (cc167) was identified in seven carrier strains from contact persons from three regions of Kazakhstan during years 2017–2018 (Table 1, S1 Table). These strains are assigned to serogroup Y and have identical PorA subtypes (P1.5–1,10–8) and FetA (F1-3). Seven strains with ST-2146 (cc198) from contact persons are identical in PorA subtypes (P1.18,2–25), but differ in FetA subtypes (F5-5 and F5-70) and are completely lacking the capsular operon (cnl). MLST ST-3015, ST-3346 (cc41/44) and ST-5636 were each represented by a single strain from contact persons from Almaty, East Kazakhstan and Akmola regions respectively. Serogroups W, B, and C were identified in these strains. Three strains from contact persons from North Kazakhstan shared ST-11585 with in silico predicted PorA subtypes (P1.5–3,10–4) and FetA subtypes (F3-72), two strains were assigned to cnl and strain NmKZ-29 has an unidentifiable capsular antigen. Sixteen ST-16025-strains from contact persons belonging to MenC, were identical in PorA (P1.22,13–2) and had two FetA alleles (F3-9 and F3-10). Two IMD strains ST-16025 belonged to MenB and differed in PorA (P1.5–1.2–2 and P1.0.25–16) and FetA (F1-90 and F1-9) alleles. One ST-16027-strain belonged to MenC and had the alleles PorA P1.22,2–26 and F3-6 FetA.

### Characteristics of non-carbohydrate protein-based vaccine targets

In total, seven fHbp peptide alleles and seven NHBA peptide alleles were identified (Table 1, S1 Table). In the 16 non-invasive and two invasive strains with new ST type 16025, fHbp allele 706 was identified, which corresponds to variant 1 (Novartis classification) or subfamily B (Pfizer classification), as well as NHBA allele 20. In strains of clonal complex cc167, fHbp allele 1084 (variant 2 or subfamily A) and NHBA allele 9 were identified. fHbp allele 1166 (variant 2 or subfamily A) was identified in ST-11585, ST-3015 and ST-12790, and NHBA allele 20 was also identified, except for the last sequence type in which allele 9 was found. In seven strains with ST-2146 (cc198) fHbp allele 614 (variant 3 subfamily A) and NHBA allele 10 were found. Two strains with ST-3346 (cc41/44) and ST-5636 have an identical fHbp allele 849 (variant 2 or subfamily A) but distinct NHBA alleles 188 and 688, respectively. fHbp alleles 34 and 613 and NHBA alleles 6 and 96 were detected in strain with new sequence type ST-16027 and strain with highly contagious ST-11, respectively. NadA peptide (NadA-2/3) was identified only in the ST-11 strain, in the rest of the samples there was a shift in the reading frame or insertion of an IS element.

## Discussion

Kazakhstan comprises the largest country in the Central Asia and is a cross-road for flows of people across the larger Eurasia. Unfortunately, epidemiological safety in the country in relation to communicable diseases such as meningitis suffers from poor knowledge on genetic features of the circulating strains. In this study, we present the first data from Kazakhstan on the genetic diversity of invasive and non-invasive strains of *N*. *meningitidis*. We used contemporary approach of whole-genome sequencing to study genetic and phenotypic characters. The WGS allowed us to identify two new MLST sequence types, one of which was found in carrier and invasive strains and the other only in an invasive strain. Despite the study has limitations given the amount of strains available for WGS, the collected data are important because the found antigenic diversity will have implications for the selection of vaccines and currently warrants to undertake measures from regulatory authorities to increase the collecting of cultures.

Strains were collected from five regions of Kazakhstan. Nine (24.2%) non-invasive strains did not have a capsular operon. Among non-invasive strains in the world, the proportion of cnl (operon-less) varies from 16 to 47%, these are common in several genetic clones of subtypes cc53, cc1117, cc192, cc845cc198 and cc1136 [18–20]. Invasive cnl strains belong to genetic clones cc192, cc198 and cc845, whereas cc53 are generally associated with the carrier state [21, 22]. In our study, seven out of nine cnl were assigned to cc198, and the remaining two strains to ST-11585. In this study, more than 51% of non-invasive strains have been assigned to MenC by genogrouping. The remaining non-invasive strains are represented by Y (16.2%), B (5.4%) and W (2.7%) serogroups. Our results are in good concordance with the results of serogrouping which shown 54% MenC in 2016–2018.

In silico MLST7 typing showed that Kazakhstan strains belong to a variety of sequence types. We identified ten STs, two of which were not previously registered in the PubMLST database. New sequence type ST-16025 (6-125-6-26-58-178-347) was identified in 18 out of 41 strains. Of this new sequence type, two invasive strains were isolated in samples from the Akmola region, with a time lag of one year; and 16 strains were obtained from contact persons in samples from the Turkestan region, collected in December 2017. Also, unexplained remains the fact that the difference between the invasive strains and carrier strain complex exceeds 2000 SNPs. Most of these differences correspond to clusters of SNPs as evaluated using Gubbins [17] and are presumably the result of horizontal gene transfer events in agreement with current knowledge [23, 24]. On the contrary, studied carrier strains appeared to be genetically homogeneous, having differences only ~60 SNPs from each other. Invasive and non-invasive strains differ in serogroups (B or C), which is a consequence of recombination. This type of recombination is more often observed between serogroups B, C, W and Y, since their capsular polysaccharide contains sialic acid. This participates in a mechanism to evade the host’s immune system [25, 26]. New sequence type ST-16027 (9-6-9-9-249-6-9) was found in one strain; more strains need to be analyzed to estimate precisely the prevalence of this lineage. All MenY strains belong to cc167, the proportion of which increased significantly in 1992–1996 in the United States, then in Latin America and some European countries [27]. An invasive strain from the hypervirulent cc11 MenW was found in cases from the Akmola region. MenW cc11, also called Hajj lineage, was first discovered in 2000 among pilgrims who returned from Mecca. Subsequently, there was an increase in this lineage among IMDs in Europe, Australia, New Zealand, the Asia-Pacific region, and also recently in China and Russia [28–31].

In a global road map from the World Health Organization on defeating meningitis, vaccination is seen as a key strategic component of the prevention of bacterial meningitis [32]. In Kazakhstan, vaccination against *N*. *meningitidis* is neither mandatory nor widely used, despite there are active proponents in the local medical community. The main hurdle for developing a national vaccination program has been the lack of knowledge of antigenic features of predominantly circulating strains, given the current absence of a multivalent vaccine for all existing serogroups. Licensed vaccines are bivalent (A, C) or tetravalent (A, C, Y, W) polysaccharide-antigen vaccines. There are also monovalent serogroups A and C and tetravalent A, C, W and Y-polysaccharide-protein conjugate vaccines [33]. The latter generate T-cell-dependent responses, which have many immunological advantages over polysaccharide vaccines, as well as inhibit transmission and reduce colonization of the nasopharynx [34, 35]. It was reported that an antigenic similarity of the group B meningococci capsule with the fetal nervous tissue results in a low immunogenicity and the risk of autoimmunity in vaccinated people, underscoring the need in a discovery of alternative antigenic components [36, 37]. Outer membrane proteins PorA, fHbp, NHBA, and NadA were considered as the main vaccine candidates. However, their use is hindered by their high genetic diversity. For example, more than 20 types of PorA were identified in the United States alone [38]. Characterization of fHbp from various strains of *N*. *meningitidis* made it possible to identify two subfamilies (A and B) or three variants (dubbed 1, 2, 3). Subfamily A fHbp is equivalent to variants 2 and 3, while subfamily B fHbp is equivalent to variant 1. The WGS of invasive and non-invasive strains of *N*. *meningitidis* is especially valuable as it makes possible to determine vaccines with the greatest homologous-antigens coverage for the population. Two vaccines against serogroup B are currently licensed: Bexsero (4CMenB) from GSK Vaccines (formerly Novartis) which includes four recombinant proteins NadA and Por NHA P1.4, and fHbp (peptide 1.1); and Trumenba (rLP2086) from Pfizer with lipidated rFHbp from two subfamilies (A and B). WGS allows controlling post-vaccination changes in the population of circulating strains. This first WGS study of *N*. *meningitidis* strains circulating in Kazakhstan will have consequences for introducing the vaccination to fight meningitis in Kazakhstan, with regard to the selection of vaccines. For example, the 4CMenB vaccine can provide protection against two invasive serogroup B strains, since they have peptide variant 1, and NHBA peptide No.20 which may cross-react with NHBA peptide No.2 [39]. However, the non-invasive serogroup B strain cc41/44 will not be covered by the 4CMenB antigenic activity. Non-invasive MenC strains ST-16025 and MenW cc11 will be covered by the 4CMenB vaccine because of the presence of fHbp and NHBA peptide No.20. In more than 97% of our strains, the NadA peptide is damaged by an IS-element insertion or frameshift mutation, making the NadA component in the 4CMenB vaccine irrelevant for Kazakhstan’s strains. Mutational changes in NadA are often seen in *N*. *meningitidis*, including urogenital strains [40, 41].

## Conclusions

This study is the first report of the genetic diversity and predicted antigenicity of *N*. *meningitidis* strains in Kazakhstan. Despite limitations due to the limited amount of strains which could be investigated here, the presented data illustrate diversity on the genetic and antigenic level requiring careful selection of vaccines. Our study will help regulatory authorities to change diagnostic routines because there is a need to increase the number of analyzed *N*. *meningitidis* strains to collect more data for vaccine matching. For future vaccination, it is important that clinics attempt to isolate bacterial cultures from all patients with meningitis. Wider use of WGS is a mandatory task to start before the next step to reduce meningitis in the country, which requires vaccination.

## Supporting information

S1 Table Results of genotyping of strains *N*. *meningitidis*. (XLS)
